# Predicting the Unmet Need for Biologically Targeted Coverage of Insecticide-Treated Nets in Kenya

**DOI:** 10.4269/ajtmh.2010.10-0331

**Published:** 2010-10-05

**Authors:** Abdisalan M. Noor, Victor A. Alegana, Anand P. Patil, Robert W. Snow

**Affiliations:** Malaria Public Health and Epidemiology Group, Centre for Geographic Medicine, Kenya Medical Research Institute/University of Oxford - Wellcome Trust Research Programme, Nairobi, Kenya; Centre for Tropical Medicine, Nuffield Department of Clinical Medicine, University of Oxford, Oxford, United Kingdom; Spatial Ecology and Epidemiology Group, Department of Zoology, University of Oxford, Oxford, United Kingdom

## Abstract

In some countries the biological targeting of universal malaria prevention may offer optimal impact on disease and significant cost-savings compared with approaches that presume universal risk. Spatially defined data on coverage of treated nets from recent national household surveys in Kenya were used within a Bayesian geostatistical framework to predict treated net coverage nationally. When combined with the distributions of malaria risk and population an estimated 8.1 million people were not protected with treated nets in 2010 in biologically defined priority areas. After adjusting for the proportion of nets in use that were not long lasting, an estimated 5.5 to 6.3 million long-lasting treated nets would be required to achieve universal coverage in 2010 in Kenya in at-risk areas compared with 16.4 to 18.1 million nets if not restricted to areas of greatest malaria risk. In Kenya, this evidence-based approach could save the national program at least 55 million US dollars.

## Introduction

Priority setting and resource allocation for disease control requires rational decisions on the geographic distribution of the populations at risk, the control interventions most appropriate to meet their health needs, and the existing levels of intervention coverage within these areas. Rational decision making for malaria is hampered because the disease burden is greatest in predominantly low income countries coincidentally plagued by weak health information and planning systems.^1^ Recent advances in high-resolution digital maps of malaria risk^2^ and population distribution^3^ provide new opportunities to identify populations at risk to guide global and regional malaria resource allocation.^4^,^5^ However, effective national resource allocation requires higher resolution malaria risk and population mapping congruent with equivalent maps of existing levels of intervention coverage. Across sub-Saharan Africa (SSA) insecticide-treated nets (ITN), notably long-lasting insecticidal nets (LLIN), remain the most widely used intervention for malaria prevention.^6^ During the early stages of ITN scale up, it was operationally easier to plan the distribution of bed nets because coverage was universally low, < 3%,^5^ and the main goal was to improve coverage to 60% of children < 5 years of age and pregnant women. Very few countries by 2007 had reached 60% coverage of ITN,^5^,^7^ and in 2008 the World Health Organization (WHO) released a call for universal coverage.^6^ In this study, universal coverage with ITN is interpreted not to mean protecting everyone living within each malaria endemic country. Rather it is presumed to be the protection of all age groups living in areas where the risk of infection merits wide scale distribution of ITN and is more cost-efficient than other methods of vector control. This critical biological distinction demands knowledge of the spatial distribution of malaria risk. Here, a Bayesian geostatistical model has been developed to predict the distribution of ITN coverage across Kenya, which when combined with modeled distributions of population and malaria risk allow for the estimation of a targeted unmet need to rapidly reach a biologically effective universal coverage of treated nets.

## Methods

### Scaling up of ITNs in Kenya and assembly of coverage data.

Since 2002 Kenya has adopted a range of ITN delivery modalities including the promotion of commercial sector subsidized net sales, the distribution of heavily subsidized and later free ITN through public health facilities to children and mothers attending antenatal services, the 2006 mass campaigns of free LLIN distribution and the 2008 net retreatment campaigns.^8^,^9^

To estimate the current level of ITN coverage, nationwide data from sample household malaria surveys undertaken since the last mass free-distribution campaign in 2006 were identified. Four surveys have been conducted since 2006 that recorded information of ITN coverage among all ages. However, the data from the Demographic and Health Survey undertaken between November and February 2008–9 were unavailable in the public domain at the time of the analysis. The remaining three surveys^10^–^12^ are summarized in Table 1. Across all surveys all-age individual level data were assembled with information on the location of households mapped using global positioning systems (GPS). These data were then aggregated by cluster to capture information on the survey location, sample size, and the sum of persons who reported to sleep under an ITN the night before the survey. Our definition of universal coverage assumes that all household members should be protected by an LLIN.^13^,^14^

### A Bayesian geostatistical model of ITN coverage for Kenya.

Bayesian geostatistical techniques^15^ allow data from point locations such as household clusters to be predicted through space to generate continuous maps with appropriate measures of uncertainty. Such techniques rely, however, on the presence of spatial autocorrelation in the variable of interest and this was tested for in the cluster level ITN/LLIN coverage data using empirical variograms [Supplementary Information (SI) 1, available at www.ajtmh.org]. A Bayesian generalized linear geostatistical model was then used to predict a 1 × 1 km map of ITN coverage for 2009. This model included two important previously documented predictors of ITN use in Kenya; urbanization and distance to ITN distribution points,^16^ described in more detail in SI 1. The spatial model was implemented in two parts starting with an inference stage in which a Markov chain Monte Carlo (MCMC) algorithm was used to generate samples from the joint posterior distribution of the parameter set and the spatial random field at the data locations. This was followed by a prediction stage in which samples were generated from the posterior distribution of the proportion of household members using an ITN/LLIN the night before the survey at each prediction location on the 1 × 1 km grid. The underlying assumption of the model was that the probability of ITN coverage at any survey location could be modeled as a continuous function of the location of the survey, modified by the two predictor variables and modeled as a transformation of a Gaussian random field. Details of the Bayesian geostatistical models are presented in SI 1.

To test the predictive accuracy of the output ITN coverage model, a geographically representative validation dataset,^17^,^18^ representing 10% of the overall data, was selected and the Bayesian geostatistical model was then implemented in full using the remaining 90% of data. The mean error, which is a measure of the bias of predictions and mean absolute error, which is a measure of overall precision were computed by comparing the predicted and the actual coverage at the validation locations.

### Estimating the biologically targeted need of LLINs in Kenya in 2010.

Data from randomized controlled trials show significant population-attributable benefits of treated bed nets among populations exposed to moderate to high levels of stable *Plasmodium falciparum* transmission, i.e., where infection prevalence is ≥ 5%. Although the use of a treated net by individuals living in areas of traditionally low parasite prevalence may provide individual protection, the cost-benefits of universal coverage remain uncertain and inconclusive.^20^,^21^ A recently produced Bayesian geostatistical 1 × 1 km grid map of malaria infection prevalence in Kenya for the year 2009, developed from empirical data from 2,682 survey locations and climatic and ecological covariates,^22^ was used to identify areas where there was less than 99% confidence that a given child in the 2–10 year age group will be uninfected; that is, where the posterior mean of *Pf*PR_2–10_ ≥ 1%. Adopting a conservative approach, these areas were considered a priority for the distribution of treated nets.

To estimate the populations living at the two different malaria risk classes, population counts from a high-resolution (100 × 100 m) population map of Kenya were extracted. This population map was developed from a combination of Radarsat-1 and Landsat Enhanced Thematic Mapper satellite imagery, land cover maps and the distributions of populations in over 40,000 census enumeration polygons.^3^ The resulting high-resolution map represented estimated population distribution in Kenya for the year 2000 and the raster population surface was projected to 2010 using provincial inter-censal growth rates from the 1999 national census.^23^

The ITN/LLIN coverage and malaria risk maps were resampled to 100 × 100 m resolutions to match that of the population distribution map. These maps were then separately overlaid on the population distribution grid to construct three-dimensional maps showing the number of people by ITN/LLIN coverage or malaria risk class using ArcScene 9.3 (ESRI Inc., New York). The intervention coverage map was then used to derive the proportion of unprotected population calculated as 1—predicted proportion of ITN/LLIN coverage at each pixel, which was then multiplied by the population at that location to generate a grid surface of the distribution of unprotected people. This was then combined with the distribution of malaria risk to identify the biologically defined needs of communities located in areas of *Pf*PR_2–10_ ≥ 1%. To control for underlying population and to ensure optimal targeting of LLINs, areas where population density was < 1 person per km^2^ were masked. Consequently, all areas where mean *Pf*PR_2–10_ was ≥ 1% and where population density was also ≥ 1 person per km^2^ were considered priority areas for the future distribution of LLINs in Kenya. The number of unprotected people living in this LLIN priority area in 2010 was then extracted using ArcGIS 9.2 *extraction* tools. The mean number of people sharing a single bed was computed from the data assembled in four sentinel districts that were representative of the broad malaria ecology in Kenya^8^ and was used to estimate LLIN needs for 2010. As a test of sensitivity, the predicted ITN map was reduced by one standard deviation below the mean and the resulting higher value of LLIN need was compared with the estimate derived on the basis of the predicted mean coverage.

## Results

### ITN coverage.

**Figure 1.**
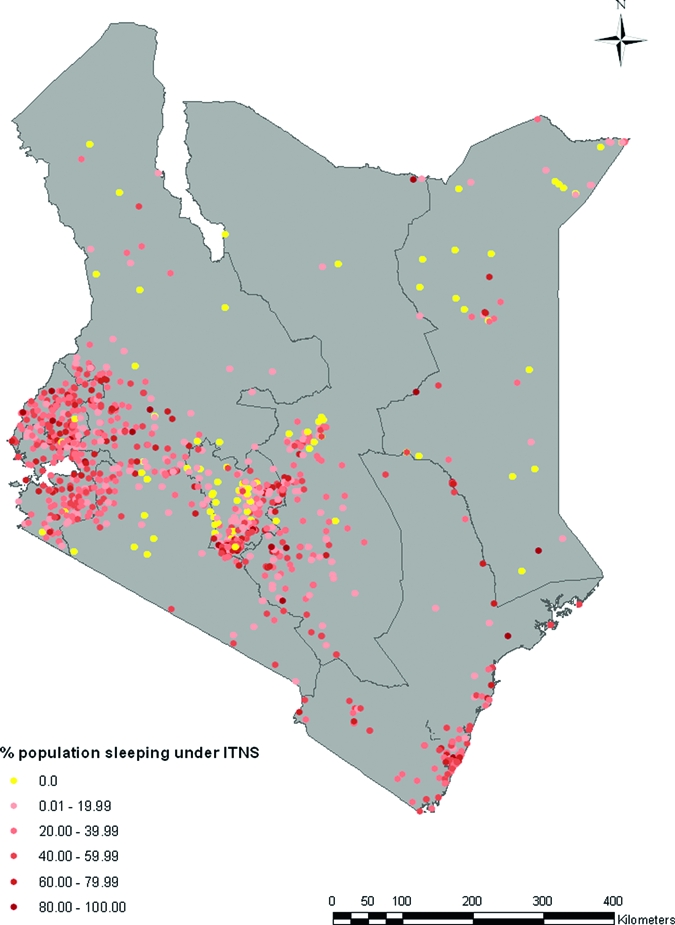
The distribution of the community survey locations showing the reported insecticide-treated nets (ITN) coverage among all ages from the MIS 2007 (*N* = 199); PSI-TRaC 2007 (*N* = 280); and the FSD 2009 (*N* = 646) surveys.

**Figure 2.**
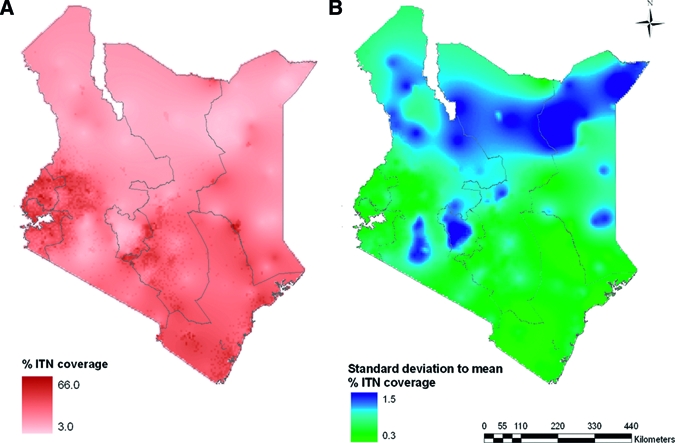
(**A**) 1 × 1 km spatial resolution Kenya map of the distribution of predicted posterior mean insecticide-treated nets (ITN) coverage among all ages in 2009; (**B**) 1 × 1 km map of the number of standard deviations from the posterior mean ITN coverage in 2009. Large values of standard deviation from the mean indicate wider confidence intervals and high uncertainty around the predictions at each 1 × 1 km grid.

A total of 1,125 ITN coverage cluster random sample surveys covering 81,964 individuals were assembled (Table 1). Aggregated ITN coverage was 35% among all ages across all surveys and varied little between surveys: 39% in July 2007, 32% in September 2007, and 33% by March 2009. The spatial distribution of the combined survey data is presented in Figure 1 and shows that observed ITN/LLIN coverage among all ages was highest in the western, coastal, and central regions of the country. The output of the Bayesian geostatistical model is shown in Figure 2A where according to the holdout sample (*N* = 113) the model had a mean error of −0.2% and a mean absolute error of 0.5%. The standard deviations from the mean predicted ITN coverage (Figure 2B) ranged from 0.3 to 1.5 standard deviations with the highest standard deviations in the northern part of the country where coverage data were sparsely distributed. From the modeled ITN/LLIN coverage shown in Figure 2A, the maximum posterior predicted mean coverage ranged from 3% to 66%, with ITN coverage in the northern part of the country generally less than 10%.

### Modeling the spatial estimates of LLIN need for 2010.

**Figure 3.**
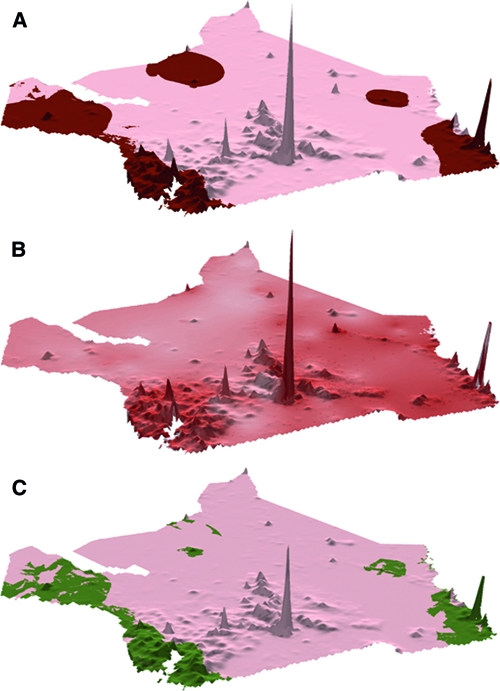
A 100 × 100 m spatial resolution three-dimensional map of population distribution in Kenya 2010 against: (**A**) malaria risk presented as modeled age-standardized *Plasmodium falciparum* parasite prevalence (*Pf*PR_2–10_) of < 1% (pink) and ≥ 1% (dark red); (**B**) the predicted posterior mean insecticide-treated nets (ITN) coverage. The darker the color the higher the predicted ITN coverage; and (**C**) areas of priority for the distribution of long-lasting insecticidal nets (LLIN) defined as those where the posterior mean malaria risk was ≥ 1% *Pf*PR_2–10_ and population density was ≥ 1 person per km^2^ shown in green. Areas shown in pink are those where *Pf*PR_2–10_ was < 1% or population was < 1 person per km^2^ and were regarded as not priority for LLIN distribution because of the low overall biological risk of infections. The three-dimensional maps were generated using Arcsence 9.3 (ESRI Inc., New York).

For visual purposes the three-dimensional 2010 population weighted distribution of the two malaria risk classes (*Pf*PR_2–10_ < 1% and ≥ 1%) are shown in Figure 3A. The map shows that the densely populated areas of the central region, including the city of Nairobi and other major towns, and the sparsely populated eastern and northern parts of the country fall in the lowest malaria risk class. The risk class ≥ 1% *Pf*PR_2–10_ covers the densely populated western highland areas, moderately populated areas on the Indian Ocean coast, and pockets north of the country. This class also covers the densely populated Lake Victoria region and small foci close to the Tanzanian border along the coast. The ITN/LLIN coverage posterior predicted map shown in Figure 2A was resampled to 100 × 100 m resolution against population density (Figure 3B). Finally, Figure 3C shows the population densities of those unprotected by either an ITN or LLIN across Kenya projected to 2010 in accordance with a single biological priority of areas where infection prevalence is predicted to be ≥ 1% and population density of ≥ 1 person per km^2^. Areas where risk was < 1% *Pf*PR_2–10_ and those where population density was < 1 person per km^2^ were considered not a priority for LLIN distribution as they were regarded to be biologically at low risk. Approximately 15.2 million people, 37.5% of Kenyans in 2010, were predicted to live in areas regarded as a priority for targeting LLINs of whom 8.1 million (53.3%) were predicted to be unprotected with a treated net in 2010 (Table 2).

Assuming an average of 1.9 people per bed it is estimated that ~4.3 million LLINs are required to reach universal coverage of protection in the high risk areas in 2010 (Figure 3C). Recognizing the need to replace ~32% of nets that are not LLIN (derived from 2007 survey data) a combined unmet and replacement need of 5.5 million LLIN in 2010 is predicted. These estimates represent 34% of the requirements defined if universal coverage were computed for all of Kenya independent of biological risk (Table 2). To provide some measure of uncertainty the population extractions were repeated using one standard deviation below the posterior prediction of the mean net coverage, thereby increasing the number of LLIN required. This interval of uncertainty is modest, about 0.8 million above estimates based on the mean, for the biologically targeted areas given that these are predominantly high population areas and with the best model predictions because of high data inputs for both malaria risk and ITN/LLIN coverage models.

## Discussion

Calls for universal coverage of interventions that prevent malaria infection^6^ recognize the importance of protecting all members of a community to maximize health impacts through systematic reductions in transmission intensity.^13^ They also, however, come at a time when financial resources for international health may be constrained by the global economic crisis. Targeting resources to reach universal coverage to those communities most likely to benefit from such a strategy would make both public health and economic sense. Across most malaria endemic countries the intensity of *P. falciparum* transmission is unevenly distributed^2^ and more imaginative selection of interventions tailored to the needs of sub-national malaria risk must become a priority to avoid wastage and inappropriate use of both prevention and disease management tools. Recognizing that “one size does not fit all” for the future of malaria control while important, demands effective tools to help national malaria programs to operationalize this concept.

Kenya is a good example of the over distribution of both malaria risks and human settlement (Figure 3A). As intervention coverage begins to escalate through mass free distribution, it becomes increasingly important to review progress against biological, population-weighted targets of universal coverage rather than national aggregate targets. Here, geostatistical Bayesian models previously used to predict malaria risk have been adapted to predict ITN/LLIN coverage. Under the reasoned assumption that future investment in LLIN distribution should be to areas of substantial transmission, classified here as a parasite prevalence ≥ 1%, we estimate that 8.1 million people lived in these areas in 2010 and are unprotected by either an ITN or a LLIN. To replace existing ITNs with LLINs and ensure universal coverage of those unprotected would require an estimated 5.5 million LLINs in 2010. These areas are also sufficiently constrained spatially to make efforts to target sub-national campaigns of free distribution, at possibly household levels, easier. Such a concerted effort would rapidly reach coverage levels, so far almost unrecorded in much of Africa with exceptions of areas in Zanzibar and Madagascar.^5^ Using the ITN/LLIN coverage estimates reduced by one standard deviation of the mean would provide a modest upward margin of uncertainty in this prediction of an additional 0.8 million nets.

An alternative to this approach is to continue to presume universal coverage independent of malaria risk. Under such a scenario the models predict that approximately 16.4 million nets would be required to reach universal coverage in 2010, including inaccessible populations in areas of very low malaria risk. This figure is closer to the number of LLIN requested by the Ministry of Health during the ninth round of funding requests to the Global Fund for acquired immunodeficiency syndrome (AIDS), tuberculosis (TB), and Malaria in 2009 (11.3 million LLINs to reach 80% coverage in Kenya by 2013, unpublished data) than our estimate of need. The cost difference between a biologically targeted and a national coverage approach is substantial, at least 55 million United States dollars (US$) for commodities alone, assuming the current price of about 5 US$ per LLIN, highlighting the benefits of a more effective spatial targeting of intervention delivery based on existing coverage, malaria risk and population settlement.

To the best of our knowledge this is the first attempt to use geostatistical models of ITN/LLIN coverage at a spatial resolution congruent with existing malaria risk and population maps to predict unmet needs. These were possible because the cluster descriptions of ITN/LLIN use showed spatial autocorrelation, clusters closer together in space were more related in their coverage than those further apart. This spatial structure in intervention coverage data offers important possibilities for modeling the spatial distribution of net use from other national sample surveys conducted across Africa,^24^–^26^ particularly in countries that share similar properties to Kenya with respect to population settlement and malaria risk. The estimated use of LLIN need presented in the analysis, however, does not account for the depreciation of LLIN that were in use at the time of the national surveys.

A conservative criterion of *Pf*PR_2–10_ < 1% was used as the threshold to define inappropriateness for universal coverage of LLIN. This is not based on any empirical cost-effectiveness modeling as the recommendations for biological targeting of LLIN remain unclear. However, it is argued here that this represents a sensible criterion for communities that historically live under these conditions and will have a low disease incidence.^27^ Furthermore, *Pf*PR_2–10_ of 1% has been proposed as a benchmark for countries to decide whether to migrate from sustained control to a strategy that focuses on elimination as the next goal.^28^,^29^ However, a word of caution is offered in the use of this criterion: for Kenya the map of 2009 malaria risk distribution largely included all areas that were historically of low parasite prevalence but as new infection data emerge and more communities are classified as less than 1% infection prevalence because of scaled intervention, different requirements for LLIN are necessary. For communities moving to a situation that supports a *Pf*PR_2–10_ of < 1% because of LLIN scale up, maintaining universal LLIN coverage will be absolutely necessary to prevent rebound and thus a new parameter will be required to define historically low *Pf*PR_2–10_ < 1% and “controlled” *Pf*PR_2–10_ < 1%. This will require a more dynamic malaria risk map to capture transitioned communities.

As countries in Africa aim for universal protection with LLIN over the next 5 to 10 years more effective sub-national planning for maximal health benefits will necessitate more informed population, risk, and coverage mapping. This should not only serve to reduce wastage of limited resources but be used as the more appropriate measure of progress. Providing national estimates of LLIN coverage among children < 5 years of age, the current standard bench mark of international monitoring must be revised. Bayesian geostatistical modeling of national, household sample survey data has a utility beyond mapping malaria risk to guide the future of resource allocation and measuring its success.

## Supplementary Material

Supplementary Information

## Figures and Tables

**Table 1:** Insecticide-treated nets (ITNs) coverage data summary overall and by source

	MIS*	PSI†	FSD‡	Total
Date	July 2007	September–October 2007	January–March 2009	
Number of clusters	199	280	646	1,125
Number of urban clusters	36	140	181	357
Number of households	6,953	4,057	6,600	17,610
Number of respondents	31,294	18,183	32,487	81,964
Mean (min, max) people per cluster	157 (78, 263)	65 (15, 131)	51 (13, 102)	73 (13, 263)
Number (%) of persons sleeping under a net	12,606 (40.3)	8,008 (44.0)	14,187 (43.6)	34,801 (42.5)
Number (%) of persons sleeping under an ITN	12,144 (38.8)	5,746 (31.6)	10,734 (33.0)	28,624 (34.9)

* The malaria indicator survey (MIS) of 2007 did not include Nairobi province.^10^

† The Population Services International – Tracking Results Continuously (PSI-TRaC) survey of 2007 survey did not include North Eastern province.^11^

‡ Financial Services Deepening (FSD) Kenya, a microfinance organization, undertook a national household survey of access to financial services in rural and urban communities.^12^ Although the FSD survey was not focused on health or malaria additional questions on net/insecticide-treated nets (ITN) use by household members of all ages were included on recommendation by the authors.

**Table 2:** Kenya 2010 estimates of population protected/not protected with insecticide-treated nets (ITNs) and long-lasting insecticidal nets (LLINs) and the number of LLINs required for universal coverage presented according to whether data were from targeted areas where LLIN distribution was a priority (age-standardized *Plasmodium falciparum* parasite prevalence (*Pf*PR_2–10_) of ≥ 1% and population density of ≥ 1 person per km^2^) and overall

	Malaria risk targeted	Not targeted
	Using predicted mean (one standard deviation below mean) ITN coverage¶	Using predicted mean (one standard deviation below mean) ITN coverage¶
Total projected population (millions) in 2010	15.2	40.5
Total projected population (millions) protected with ITNs in 2010	7.1 (4.7)	17.0 (9.5)
Total projected population (millions) protected with LLINs in 2010†	4.8 (3.2)	9.4 (5.2)
Total projected population (millions) protected with ITNs but not LLINs in 2010	2.3 (1.5)	7.6 (4.3)
Total population (millions) NOT protected with ANY treated nets in 2010	8.1 (10.5)	23.5 (31.0)
Total projected population (millions) that need LLINs in 2010‡	10.4 (12.0)	31.1 (35.3)
Mean number of persons sharing a bed§	1.9	1.9
Number of LLINs in millions needed for universal coverage in 2010	5.5 (6.3)	16.4 (18.1)

† Percentage of treated nets that were LLIN of 68% in the targeted areas and 55% overall were derived from the 2007 national surveys.^10^ These were used to compute the number of people that were protected with ITNs of the LLIN type in 2010.

‡ Total number of individuals who needed protection with LLIN in 2010 was computed from the sum of the total number of people not protected with any nets in 2010 and the number who were protected with a treated net that was not an LLIN.

§ Mean number of persons sharing a bed net of 1.9 was derived from household surveys done in 72 communities in four districts in 2006 covering the diverse malaria ecology in Kenya.^8^

¶ Estimates in brackets represent those that are based on the number of people who were not protected with LLIN when the mean predicted ITN coverage was reduced by one standard deviation to provide an upper limit of LLIN need.
