# Supplementary material for: Predicting the Unmet Need for Biologically Targeted Coverage of Insecticide-Treated Nets in Kenya
Source: Am J Trop Med Hyg. 2010 Oct 5;83(4):854–60. doi: 10.4269/ajtmh.2010.10-0331 (PMC2946756; doi:10.4269/ajtmh.2010.10-0331)
Supplement: Supplementary Information [file SD3.pdf]

SUPPLEMENTARY INFORMATION (SI) 1

Details of the semi-variogram of insecticide net treated (ITN) coverage data, analysis of predictors of ITN coverage and the details of the Bayesian geo-statistical model procedures are presented here.

1.1. Semivariogram of the ITN coverage data. The variogram or semivariogram is a graphical summary of spatial autocorrelation structure in the data. The semivariogram was calculated using the *variogram* function and an exponential model was fit using *variofit* function in R (R version 2.10.1, the R foundation for Statistical Computation, http://www.r-project.org/). Only data of sample size of ≥ 40 persons (N = 942) were used to construct the semivariogram. This sample size restriction was imposed to minimize the effect of random variations inherent in small samples.[1,2] The semivariogram of the data showed spatial autocorrelation in the combined ITN coverage data of up to 2 decimal degrees or 222 km at the equator (Figure S1).

1.2. Analysis of the predictors of ITN coverage. In Kenya, urbanization,[3] physical access to sources of ITNs,[3] and poverty[3,4] have all been shown to be determinants of ITN coverage. For these covariates to be used for the prediction of ITN coverage, however, they must be universally available at similar spatial resolutions for both the survey locations and across the prediction surface. For this study only urbanization and distance to ITN sources met this criterion. Poverty data for Kenya were only available for relatively large administrative areas such as locations and constituencies and were too coarse for consideration as mapped covariates in this study.

1.2.1. Urbanization. To define urban extents in Kenya the 1999 national census urban-rural definition of enumeration areas (EA) was used.[5] An enumeration area was defined as including part of a village, a whole village, or group of villages that are usually not more than 100 households (circa 500 people).[5] The EA maps for 67 of 69 districts in Kenya were either obtained from the Kenya National Bureau of Statistics or were digitized in-house from 1:50,000 topographical maps and each EA was classified as urban or rural. The two districts where EA data were not available were Nairobi and Mombasa, both of which were exclusively urban. The EA map was gridded into a 1 × 1 km surface and the urban/rural classifications were extracted to each survey location using ArcGIS 9.3 (ESRI Inc., New York) *extraction* tools.

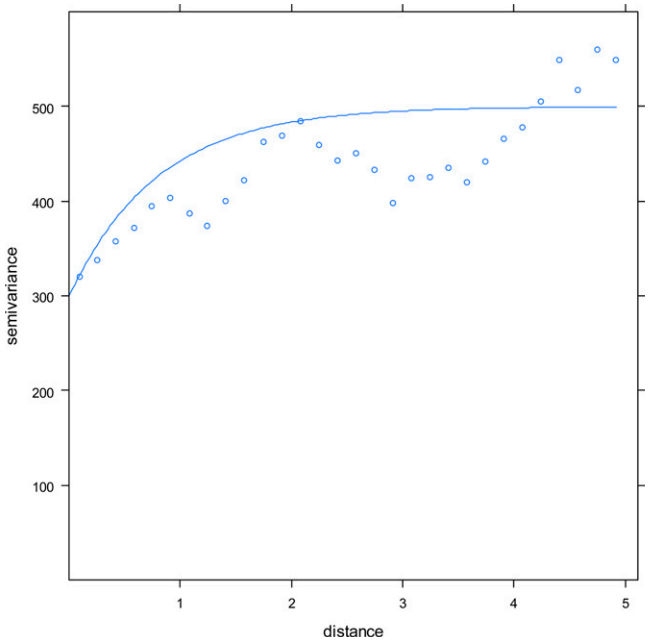

FIGURE S1. Semivariogram and model fit for distribution of *the ITN coverage* data (N = 942 clusters). The X axis shows distance in degrees of both latitude and longitude, whereas the Y axis shows the semivariance.

1.2.2. Distance to sources of ITN. A list of all antenatal health facilities where ITNs were distributed to pregnant women and mothers of young children either as highly subsidized or at no cost approaches for the entire period of distribution were provided by Population Services International (PSI). For this study, those facilities that distributed PSI bed nets from May 2005 to February 2009 were selected, corresponding to the month when the LLINs were first introduced in the Kenya public sector to the month the FSD survey, the most recent of the national household ITN surveys, ended. The 2006 free mass campaign implemented by the Ministry of Health (MoH) used health facilities, market centers, schools, churches, and other community focal points as delivery sites for LLINs. Additional countrywide focal distribution data were extracted from the website of the Against Malaria Foundation [http://www.againstmalaria.com/Distribution_Countries.aspx]. For the districts in the North Eastern province, health facilities and villages where ITNs were distributed by the MENTOR initiative, United Nations Children's Fund (UNICEF), and the Kenya Red Cross were assembled. The health facilities, market centers, schools, and other sites that were used by the various distribution agents for either the routine or free mass distribution of ITNs were mapped using national geographic information systems databases of health service providers[6]; schools[7]; and settlements[8,9]; and a variety of smaller databases developed as part of research projects or development programs. To compute distances to the mapped distribution points, a 100 × 100 m spatial resolution Euclidean distance (km) surface to ITN sources was generated and extracted to each survey location using ArcGIS 9.3.

1.2.3. Regression analysis. The relationships of urbanization and distance to ITN distribution points to the reported ITN coverage were examined using regression analysis in Stata/SE Version 10 (Stata Corp., College Station, TX). A univariate non-spatial binomial logistic regression model was implemented for these predictors with ITN coverage as the dependent variable. The results of the univariate analyses were used to determine the independent influence of each candidate predictor on ITN coverage and identify those which qualified for inclusion in the Bayesian geostatistical model. Those with Wald's P > 0.2 were considered to have qualified for inclusion into the geostatistical model to predict ITN coverage.

The regression analysis showed that urban clusters (odds ratio [OR]: 1.85, 95% confidence interval [CI]: 1.32–2.59; P < 0.001) had significantly higher mean ITN coverage compared with rural, whereas those outside the mean distance to ITN sources (OR: 0.59, 95% CI: 0.45–0.78; P < 0.001) had lower mean ITN coverage. Both covariates met the inclusion

criterion into the Bayesian geostatistical model of $P < 0.20$ and were therefore used for predicting ITN coverage.

SI 2.1. Model overview. The underlying value of ITN coverage among all ages, ITNx_i, at each location x_i for the year 2009 was modeled as a an inverse logit transformation of a spatial structured field superimposed with additional random variation $\varepsilon(x_i)$.[10] The count of individuals sleeping under an ITN the night before survey N_i^+ from the total sample of N_i in each survey was modeled as a conditionally independent binomial variate.[11] The spatial component was represented by a stationary Gaussian process $f(x_i)$ with mean μ and covariance C, defined below. The unstructured component $\varepsilon(x_i)$ was represented as Gaussian with zero mean and variance V. Both the inference and prediction stages were coded using Python (PyMC version 2.0).[12]

Mean definition. The mean component μ was modeled as a linear function of whether the prediction location was: urban (denoted by the indicator variable $1_{ur1}[x]$) rather than rural; at distance to ITN sources of ≥ 3 km ($1_{wb1}[x]$) rather than < 3 km. The mean component was therefore defined by three parameters:

$$\mu = \beta_x + \beta_{ur1} 1_{ur1}(x) + \beta_{wb1} 1_{wb1}(x),$$

where β_x denotes the intercept. Each survey was referenced temporally using the mid-point (in decimal years) between the recorded start and end months.

COVARIANCE DEFINITION

The spatial covariance was modeled as follows:

$$\varphi \sim \text{Exponential}(.1)$$
$$\psi \sim \text{Uniform}(0, 1)$$
$$\varphi_s = \psi\varphi$$
$$\varphi_l = (1 - \psi)\varphi$$
$$\upsilon_l \sim \text{Exponential}(.1)$$
$$\upsilon_s \sim \text{Exponential}(.1)1_{\upsilon_s < \upsilon_l}$$
$$\nu \sim \text{Uniform}(.1, 3)$$
$$C(x, y) = \varphi_s \text{Matern}(d(x, y)/\upsilon_s; \nu) + \varphi_l \exp - (d(x, y)/\upsilon_l))^2$$

The Matern covariance function, used for the short-range component, includes a parameter ν that controls the degree of differentiability, whereas the exponential covariance function forces the long-range component to be infinitely differentiable.[13] To allow for spatial anisotropy and to accomodate the effect of the curvature of the earth on point-to-point separations, spatial distance between a pair of points x_i and x_j was computed as great-circle distance $d_{GC}(x_i, x_j)$ multiplied by a factor that depends on the angle of inclination $\theta(x_i, x_j)$ of the vector pointing from x_i to x_j. θ was computed as if latitude and longitude were Euclidean coordinates (on a cylindrical projection). Pairs of points for which θ was close to an unknown angle λ were relatively highly correlated. The magnitude of this 'eccentricity' was controlled by an unknown parameter ω:

$$\lambda \sim \text{Uniform}(0, 2\pi)$$
$$\omega \sim \text{Uniform}(0, .95)$$
$$d(x_i, x_j) = d_{GC}(x_i, x_j)1 - \omega^2\cos^2(\theta(x_i, x_j) - \lambda)$$

SI 2.2. Model implementation and output. Bayesian inference was implemented using Markov chain Monte Carlo to generate samples from the posterior distribution of the Gaussian field $f(x_i)$ at each data location and of the unobserved parameters of the mean, covariance function, and Gaussian random noise component.

Samples were generated from the mid-year 2009 mean of the posterior distribution of $f(x_i)$ at each prediction location. For each sample of the joint posterior, predictions were made at points on a regular 1×1 km spatial grid across Kenya. Model output therefore consisted of samples from the predicted posterior distribution of the 2009 mean ITN coverage at each grid location, which were used to generate point estimates (Figure 2A in the main text) (computed as the mean of each set of posterior samples).

REFERENCES

1. Noor AM, Moloney G, Borle M, Fegan GW, Shewshuk T, Snow RW, 2008. The use of mosquito nets and the prevalence of *Plasmodium falciparum* infection in rural South central Somalia. *PLoS One 3:* e2081.
2. Jovani R, Tella J, 2006. Parasite prevalence and sample size: misconceptions and solutions. *Trends Parasitol 22:* 214–218.
3. Noor AM, Omumbo JA, Amin AA, Zurovac D, Snow RW, 2006. Wealth, mother's education and physical access as determinants of retail sector net use in rural Kenya. *Malar J 5:* 5.
4. Chuma JM, Thiede M, Molyneux CS, 2007. Rethinking the economic costs of malaria at the household level: eidence from applying a new analytical framework in rural Kenya. *Malar J 5:* 76.
5. Central Bureau of Statistics, 2001. *1999 Population and Housing Census, Volume 1: Population Distribution by Administrative Areas and Urban Centers*. Nairobi Kenya: CBS.
6. Noor AM, Alegana VA, Gething PW, Snow RW, 2009. A spatial national health facility database for public health sector planning in Kenya in 2008. *Int J Health Geogr 8:* e13.
7. Ministry of Education, 2008. *Inception Report: Consultancy on Development of a GIS Database of Learning Institutions (School Mapping Exercise)*. Nairobi, Kenya: Oakar Services Ltd.
8. Ministry of Roads and Public Works, 2008. *Classified Digital Road Network in Kenya*. Nairobi, Kenya: Roads Department.
9. International Livestock Research Institute, 2004. *GIS Database*. Available at: http://www.ilri.org/gis/search.asp?id=372. Accessed April 20, 2010.
10. Hay SI, Guerra CA, Gething PW, Patil AP, Tatem AJ, Noor AM, Kabaria CW, Manh BH, Elyazar IR, Brooker S, Smith DL, Moyeed RA, Snow RW, 2009. A world malaria map: *Plasmodium falciparum* endemicity in 2007. *PLoS Med 6:* e1000048.
11. Diggle P, Moyeed R, Rowlingson B, Thompson M, 2002. Childhood malaria in The Gambia: a case-study in model-based geostatistics. *Appl Stat 51:* 493–506.
12. Patil AP, Huard D, Fonnesbeck C, 2010. PyMC 2.0. Bayesian stochastic modelling in Python. *J Stat Softw 35:* 4.
13. Banerjee S, Carlin BP, Gelfand AE, 2003. Hierarchical modeling and analysis for spatial data. Boca Raton: Chapman & Hall/CRC.
